# Targeting CD38 in relapsed /refractory T-cell acute lymphoblastic leukemia/lymphoma: A systematic review of daratumumab-based therapy

**DOI:** 10.1177/20406207261484097

**Published:** 2026-08-25

**Authors:** Abdulrahman F. Al-Mashdali, Mujahid O. Abdelraof, Monia Ibrahim, Mohammed Abdulgayoom, Shehab F. Mohamed, Honar Cherif

**Affiliations:** 1 Department of Hematology and Bone Marrow Transplant, National Center for Cancer Care and Research, Hamad Medical Corporation, Doha, Qatar; 2 Department of Public and Environmental Health, Faculty of Medicine, 472715 Gezira University, Wad Madani, Sudan

**Keywords:** daratumumab, T-ALL, T-LBL, relapsed/refractory leukemia, CD38

## Abstract

**Background:**

Relapsed/refractory (R/R) T-cell acute lymphoblastic leukemia/lymphoma (T-ALL/T-LBL) has poor outcomes and limited salvage options. Objectives: To evaluate the efficacy and safety of daratumumab-based therapy in R/R T-ALL/T-LBL.

**Design:**

Systematic review of observational reports.

**Methods:**

Sixteen reports including 34 patients treated with daratumumab-based regimens were reviewed. Baseline characteristics, treatment patterns, response, MRD status, survival, allogeneic HSCT, and safety were descriptively synthesized.

**Results:**

Complete remission was reported in 30/34 patients (88.2%), partial remission in 2/34 (5.9%), and no response in 2/34 (5.9%). Among complete responders with reported MRD status, 24/29 (82.8%) achieved MRD negativity. Median PFS was 10 months (range, 0.4–45), and median OS was 10 months (range, 1.5–45), although many values reflected last follow-up. Eighteen patients (52.9%) proceeded to allogeneic HSCT; 13/18 (72.2%) were alive at last follow-up. Infusion reactions occurred in 10 patients, mostly grade 1–2.

**Conclusion:**

Daratumumab-based therapy may induce remission and facilitate HSCT bridging in selected patients, but findings remain hypothesis-generating because evidence is mainly from case reports and small series.

## Introduction

T-cell acute lymphoblastic leukemia (T-ALL) and T-lymphoblastic lymphoma (T-LBL) represent aggressive hematologic malignancies arising from precursor T-lymphocytes, characterized by rapid progression and a high risk of treatment failure.Although initial therapeutic approaches for newly diagnosed disease are generally effective, 10% to 25% of patients become refractory to or relapse after frontline therapy. Once relapse or refractoriness occurs, outcomes decline sharply, establishing relapsed/refractory T-ALL/LBL (R/R T-ALL/LBL) as a population with a particularly poor prognosis and no standardized, consistently effective salvage regimen.^1–3^ The challenges associated with R/R T-ALL/LBL are well documented. Multiple studies describe survival in this setting as unsatisfactory, with limited curative options and no consensus on optimal management.^4,5^

CD38 has emerged as a biologically relevant and potentially targetable antigen in T-ALL. Its expression is described as nearly ubiquitous, with up to 98% of patients exhibiting CD38-positive blasts and nearly 90% showing expression levels above 80%.^5–7^ Preclinical evidence from patient-derived xenograft models demonstrated that daratumumab effectively targets CD38-positive T-ALL blasts and can overcome chemotherapy resistance, providing the rationale for clinical investigation in this setting.^8^ This stability and frequency position CD38 as an appealing therapeutic target and provides a biologic rationale for exploring CD38-directed strategies in R/R T-ALL and T-LBL.

**Figure 1.**
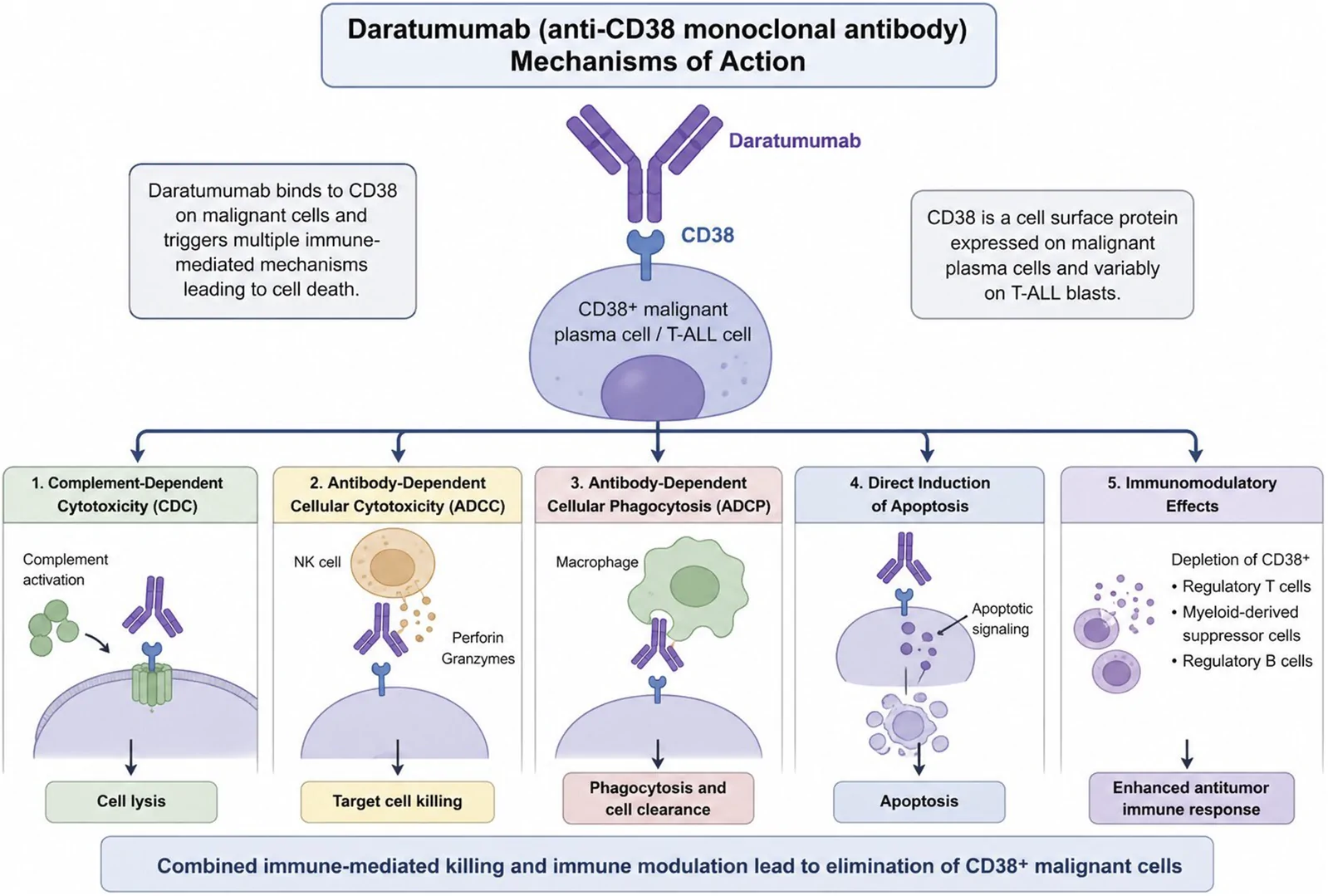
Mechanisms of action of daratumumab. Daratumumab targets CD38-positive malignant cells through immune-mediated cytotoxicity, phagocytosis, apoptosis, and immunomodulatory effects (This figure was generated with AI assistance and reviewed and approved by the authors).

Daratumumab is a human IgG1κ monoclonal antibody that targets CD38, a transmembrane glycoprotein highly expressed on plasma cells and variably expressed on leukemic blasts, including T-ALL. After binding to CD38, daratumumab induces tumor-cell killing through several immune-mediated mechanisms, including complement-dependent cytotoxicity, antibody-dependent cellular cytotoxicity, antibody-dependent cellular phagocytosis, and direct apoptosis. In addition, daratumumab may exert immunomodulatory effects by depleting CD38-positive immunosuppressive cells, thereby enhancing antitumor immune activity (Figure 1).^9^ Daratumumab has demonstrated potent activity in multiple myeloma and has generated interest for repurposing in T-ALL/LBL given the high prevalence of CD38 expression. However, clinical experience in T-ALL remains limited, consisting primarily of case reports and small case series. Furthermore, emerging phase 2 data from studies such as the DELPHINUS trial indicate that daratumumab combined with conventional chemotherapy can elicit encouraging response rates and allow effective bridging to HSCT in pediatric and young adult cohorts with R/R T-ALL/T-LBL.^3^ As a result, the full therapeutic potential, safety profile, and durability of responses to daratumumab, either as monotherapy or in combination with other agents have not been comprehensively defined.

Given these gaps, a systematic synthesis of existing evidence is needed to clarify the antileukemic activity, safety, and translational relevance of daratumumab in R/R T-ALL/LBL. Such a review will guide clinical decision-making, inform future research directions, and support the development of rational CD38-targeted strategies for this high-risk population.

## Methods

### Protocol and reporting

An a priori protocol defining the eligibility criteria, outcomes of interest, data extraction process, risk-of-bias assessment, and analytic methods was developed before data collection. The protocol was not prospectively registered; however, it is provided as Supplemental Material to enhance methodological transparency. The review was conducted and reported in accordance with the PRISMA 2020 guidelines.^10^

### Study eligibility and scope

This review evaluated real-world evidence on daratumumab use in patients with R/R T-ALL/T-LBL. Pediatric, adolescent/young adult, and adult populations were eligible. Studies were included when disease-specific data were extractable. Reports exclusively addressing newly diagnosed disease or other T-cell malignancies were not considered.

Eligible interventions included daratumumab administered via any route (intravenous or subcutaneous), as monotherapy or in combination with chemotherapy or targeted agents. There were no restrictions on dosing, schedule, or treatment line, and supportive or adjunctive therapies were permitted. No comparator was required, and single-arm studies were eligible.

Studies were required to report at least one clinical efficacy, survival, or safety outcome. Outcomes of interest included remission status, MRD assessment, survival endpoints, transplantation outcomes, CD38 expression, and treatment-related adverse events.

Eligible study designs comprised observational cohorts, case series, and individual case reports. Conference abstracts were included if they have enough data. Interventional clinical trials, reviews, editorials, laboratory-only studies, animal studies, and reports without extractable individual-level clinical data were excluded. Only English-language publications were considered.

### Literature search and study selection

A comprehensive literature search was conducted in PubMed/MEDLINE, Scopus, and Web of Science from database inception through December 1, 2025. The search strategy combined controlled vocabulary terms and keywords related to T-ALL, T-LBL, CD38, and daratumumab. Reference lists of eligible articles and relevant conference proceedings, including ASH, EHA, and ASCO, were also screened to identify additional studies. Two reviewers independently screened titles and abstracts, followed by full-text assessment of potentially eligible articles. Any discrepancies were resolved by consensus or consultation with a third reviewer.

### Data extraction

Data were independently extracted by two reviewers using a standardized electronic form. Extracted variables included study characteristics; patient demographics and disease features; prior treatment history; daratumumab dosing, route, and combination regimens; CD38 expression; clinical and MRD responses; survival outcomes; transplantation data; and safety events, including infusion-related reactions, cytopenias, and infections.

### Quality assessment

The methodological quality of included studies was assessed using tools appropriate for the specific study design. For case reports and case series, which comprised a significant portion of the dataset, we utilized the Murad tool^11^ to evaluate domains such as selection, ascertainment, causality, and reporting. Retrospective Cohorts were assessed for risk of bias using Newcastle-Ottawa Scale.^12^ Of note, because most included studies were uncontrolled case reports or small case series, the tool was used to assess reporting quality and internal methodological completeness rather than to infer high certainty of evidence.

### Statistical analysis

Descriptive statistics were used to summarize clinical characteristics, treatment patterns, and outcomes. Continuous variables, including age, number of prior lines of therapy, and blast percentages, were reported as medians with ranges or interquartile ranges (IQR), where available. Categorical variables, including sex, cytogenetic abnormalities, response rates, MRD status, transplantation outcomes, and adverse events, were presented as frequencies and percentages.

Response rates, including complete remission (CR), partial remission (PR), and no response (NR), were calculated for the aggregate population and stratified by study type where appropriate. MRD negativity was defined according to the thresholds reported in individual studies. The proportion of patients successfully bridged to allogeneic HSCT after daratumumab was also summarized.

Survival data were extracted as reported in the original studies. Overall survival was defined as the time from daratumumab initiation to death from any cause or last follow-up, while progression-free survival was defined as the time from daratumumab initiation to documented progression, relapse, death, or last follow-up. Because the evidence consisted predominantly of case reports and small case series, survival endpoints were inconsistently reported; some studies provided OS, others provided PFS, and some reported only follow-up duration after daratumumab initiation. In selected cases, survival duration was manually estimated from published figures, timelines, or Kaplan–Meier plots when exact numerical values were not provided.

Given the heterogeneity of study designs, treatment regimens, outcome definitions, and survival reporting, formal meta-analysis and pooled OS or PFS analysis were not performed. Instead, available time-to-event values were summarized descriptively as reported survival duration after daratumumab initiation using median and range. Data analysis was performed using standard spreadsheet software.

### Ethics and data availability

Because this study synthesized published data only, institutional review board approval was not required. Extraction forms, analytic code, and pooled summary tables are available on request, and detailed results are presented in the supplemental material.

## Results

### Search strategy

**Figure 2.**
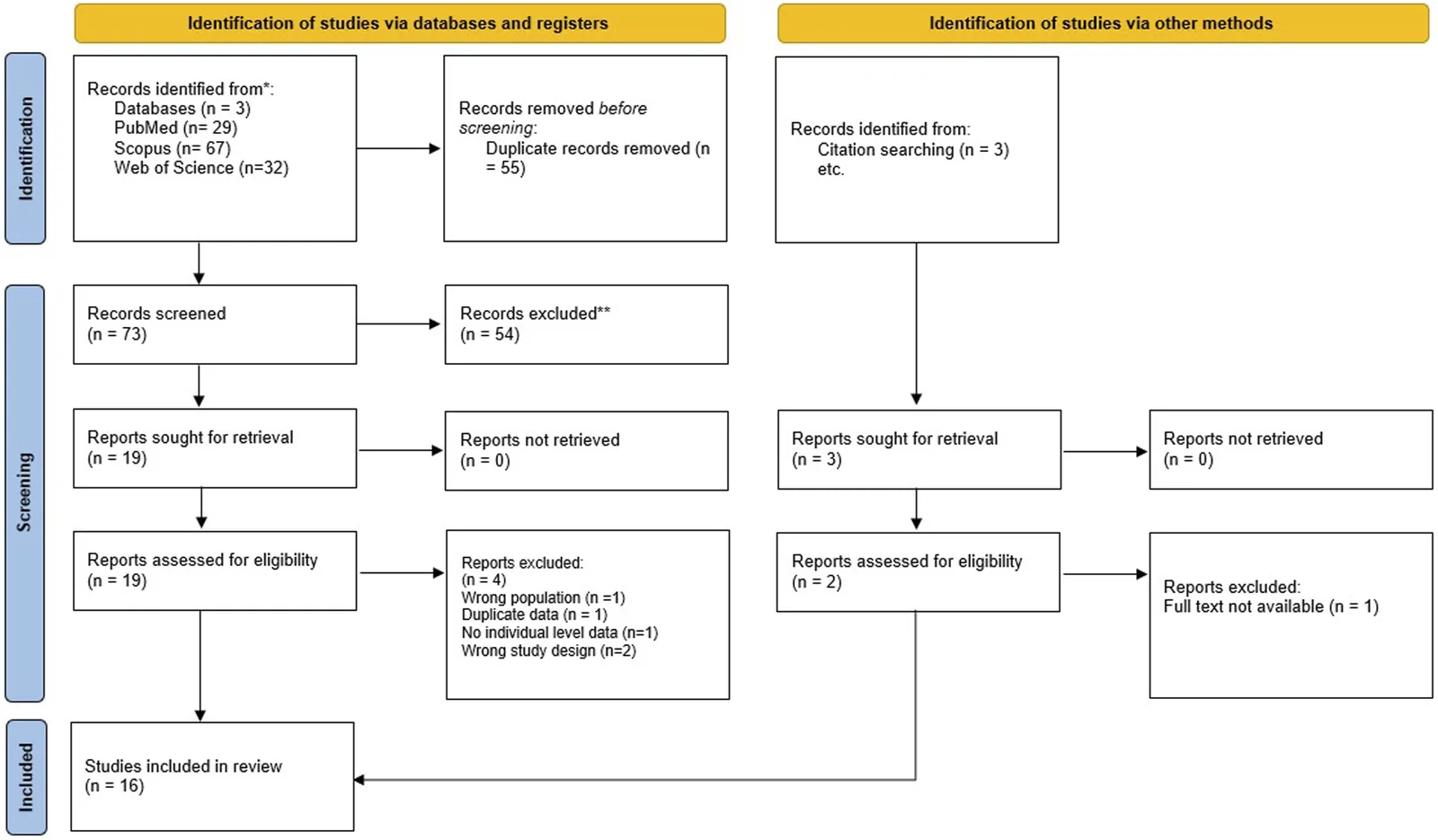
PRISMA flow diagram of study selection. Flowchart illustrating the identification, screening, eligibility assessment, and inclusion of studies in accordance with PRISMA guidelines.

A comprehensive literature search was performed in PubMed, Scopus, and Web of Science. The search identified 128 records (PubMed, n = 29; Scopus, n = 67; Web of Science, n = 32), with an additional three records retrieved through citation searching. After removal of 55 duplicates, 73 unique records underwent title and abstract screening, of which 54 were excluded. Nineteen full-text articles were reviewed for eligibility; five were excluded due to incorrect population (n = 1), inappropriate study design (n = 2), duplicate data (n = 1), or lack of individual-level data (n = 1). Two additional eligible studies were identified through reference screening. In total, 16 studies were included in the final review (Figure 2).

### Quality assessment

All included reports, except one retrospective study assessed using the Newcastle–Ottawa Scale, fulfilled the key methodological quality domains for case reports and case series, including adequate clinical description, exposure ascertainment, and outcome reporting. However, the overall certainty of evidence remained low because most studies were uncontrolled case reports or small case series, with inherent risks of selection bias, publication bias, confounding, and absence of comparator groups. Detailed results of the quality assessment are provided in Supplemental Tables 1 and 2.

### Baseline characteristics

A total of 16 studies, comprising 34 patients with R/R T-ALL/T-LBL (T-ALL; bone marrow involvement ≥20% or T-LBL; bone marrow involvement <20%), were included.^5,7,13–26^ All studies were observational, consisting primarily of single-institution case reports or small case series published between 2018 and 2025 across Europe, Asia, and North America.

Most patients were diagnosed with T-ALL (31/34, 91.2%), with three cases of T-LBL (3/34, 8.8%). The cohort consisted predominantly of adults, with one pediatric patient (1/34, 2.9%)—a 2-year-old described by Ruhayel et al.^24^ Sex was reported for 30 patients: 16 males (53.3%) and 14 females (46.7%). Sex was not reported in four cases (11.8%), all from Vakrmanová et al.^25^

Cytogenetic and molecular findings were heterogeneous. Cytogenetic abnormalities varied widely (Table 1), and most studies did not identify recurrent molecular alterations. High-risk molecular features and unusual disease biology were reported in individual cases, including a refractory patient harboring WT1, TP53, and JAK3 mutations^16^ and a patient initially diagnosed with leukemia of ambiguous lineage, later reclassified as T-ALL after relapse, who ultimately underwent two allogeneic transplants prior to daratumumab therapy.^14^ The early T-precursor (ETP) phenotype was confirmed in six patients, although reporting was inconsistent across studies.

**Table 1.** Baseline characteristics of patients treated with daratumumab for T-ALL/T-LBL.

No.	Study ID	Patient ID within study	Country	Study design	Total eligible cases in study	Diagnosis	Age	Sex	Cytogenetic/karyotype abnormality	Indication for daratumumab	ETP status	Prior systemic therapy lines	Prior systemic therapies	Prior allo-HSCT
1	Prejzner, 2023^22^	Patient 1	Poland	Case series	2	T-ALL	20	F	t (2; 14) (q21; q23)	Refractory disease	NR	1	Steroid pretreatment; cyclophosphamide; hyper-CVAD	No
2	Prejzner, 2023^22^	Patient 2	Poland	Case series	2	T-ALL	24	F	del (5) (q22q33), del (11) (q22q33)	Relapse	NR	4	hyper-CVAD/MA; methotrexate, vincristine, and doxorubicin; clofarabine and cytarabine; bortezomib with venetoclax; nelarabine	Yes
3	Vakrmanová, 2022^25^	Patient 1	Czech Republic	Case series	4	T-ALL	NR	NR	NR	Relapse	NR	1	NR	Yes
4	Vakrmanová, 2022^25^	Patient 2	Czech Republic	Case series	4	T-ALL	NR	NR	NR	Relapse	NR	1	NR	No
5	Vakrmanová, 2022^25^	Patient 3	Czech Republic	Case series	4	T-ALL	NR	NR	NR	Relapse	NR	1	NR	No
6	Vakrmanová, 2022^25^	Patient 4	Czech Republic	Case series	4	T-ALL	NR	NR	NR	Relapse	NR	1	NR	Yes
7	Molle, 2022^19^	Patient 1	Denmark	Case series	2	T-LBL	39	M	NR	Relapse	NR	1	NOPHO2008 high-risk protocol; FLAG-IDA	No
8	Molle, 2022^19^	Patient 2	Denmark	Case series	2	T-ALL	25	F	NR	Relapse	NR	2	NOPHO2008 high-risk protocol; allo-HSCT conditioning with etoposide and TBI	Yes
9	Punatar, 2021^23^	Patient 1	India	Case report	1	T-ALL	NR	F	NR	Relapsed/refractory disease	Yes	4	3+7 followed by HiDAC; salvage chemotherapy; ALL-like salvage chemotherapy; FLAG-IDA; dexamethasone, vincristine, pegylated L-asparaginase, and bortezomib	Yes
10	Fulcher, 2021^17^	Patient 1	Canada	Case report	1	T-ALL	20	F	47,XX,+6,+7 in 9/19 metaphases	Refractory disease	Yes	2	Hyper-CVAD; FLAG-IDA	No
11	Mirgh, 2019^18^	Patient 1	India	Case series	2	T-ALL	57	M	Normal	Refractory disease	Yes	1	UKALL-XII protocol	No
12	Mirgh, 2019^18^	Patient 2	India	Case series	2	T-ALL	26	M	NR	MRD positivity	Yes	2	Children’s Oncology Group 0232 protocol; Hyper-CVAD	Yes
13	Begna, 2024^13^	Patient 1	USA	Case report	1	T-ALL	45	F	TRB rearrangement at 7q34 and trisomy 9	Refractory disease	NR	4	Hyper-CVAD; liposomal vincristine and venetoclax; venetoclax with FLAG-IDA; nelarabine	Yes
14	Ruhayel, 2021^24^	Patient 1	NR	Case report	1	T-ALL	2	M	Normal	Refractory disease	NR	4	BFM 2009 protocols IA and IB; COG AALL1231 high-risk intensification blocks; nelarabine-containing chemotherapy	No
15	Bonda, 2018^14^	Patient 1	India	Case report	1	T-ALL	36	F	NR	MRD positivity	Yes	4	3+7; ALL-like induction; vincristine, etoposide, bortezomib, dexamethasone, adriamycin, and L-asparaginase; FLAG-IDA; pegylated L-asparaginase, dexamethasone, bortezomib, and vincristine	Yes
16	Ofran, 2020^20^	Patient 1	Israel	Case series	2	T-LBL	26	M	NR	Relapse	NR	3	ECOG 2993-based induction; HiDAC/mitoxantrone; nelarabine	Yes
17	Ofran, 2020^20^	Patient 2	Israel	Case series	2	T-ALL	45	F	Normal	Relapse	NR	3	ECOG 2993-based induction; mitoxantrone plus HiDAC; nelarabine	Yes
18	Castellanos, 2024^15^	Patient 1	Spain	Case report	1	T-ALL	19	M	Normal	Relapse	NR	1	Hyper-CVAD; cyclophosphamide and prednisone prephase; intrathecal methotrexate, cytarabine, and dexamethasone	No
19	Maraglino, 2025^5^	Patient 1	Italy	Case report	1	T-LBL	50	M	Normal	Refractory disease	NR	2	Hyper-CVAD plus autologous HSCT; nelarabine monotherapy for 2 cycles	No
20	Kaya, 2025^26^	Patient 1	Turkey	Case report	1	T-ALL	21	M	Normal	Refractory disease	Yes	3	HOELZER protocol; FLEND plus PEG-asparaginase; BFM 2000	No
21	Feng, 2025^7^	Patient 1	China	Case report	1	T-ALL	60	F	NR	MRD positivity	NR	1	Vincristine, daunorubicin, L-asparaginase, and prednisone induction chemotherapy	No
22	Dai, 2024^16^	Patient 1	China	Retrospective cohort	2	T-ALL	25	M	Normal	Refractory disease	NR	2	NR	No
23	Dai, 2024^16^	Patient 2	China	Retrospective cohort	2	T-ALL	52	M	NR	Relapse	NR	2	NR	No
24	Peng, 2025^21^	Aggregate cohort	China	Retrospective cohort	11	T-ALL	Median 26; range 15–44	6 M/5 F	NR	MRD positivity in 9; relapse in 2	NR	NR	Intensive multidrug chemotherapy	No

**Abbreviations:** T-ALL, T-cell acute lymphoblastic leukemia; T-LBL, T-cell lymphoblastic lymphoma; CD38, cluster of differentiation 38; del(X), deletion of chromosome X; NOTCH1, neurogenic locus notch homolog protein 1; t(X; Y), translocation between chromosomes X and Y; TRB, T-cell receptor beta; 47,XX, karyotype with 47 chromosomes (trisomy) and female sex chromosomes; +X, trisomy of chromosome X; q, long arm of a chromosome; NA, not available; allo-HSCT, allogeneic hematopoietic stem cell transplantation; auto-HSCT, autologous stem cell transplantation; BFM, Berlin–Frankfurt–Münster chemotherapy protocol; COG, Children’s Oncology Group protocol; ECOG, Eastern Cooperative Oncology Group; FLAG-IDA, fludarabine, cytarabine, G-CSF, and idarubicin; FLEND, fludarabine, etoposide, nelarabine, and dexamethasone; HiDAC, high-dose cytarabine; hyper-CVAD, cyclophosphamide, vincristine, doxorubicin, and dexamethasone; MRD, minimal residual disease; NOPHO, Nordic Society of Paediatric Haematology and Oncology; PEG-asparaginase, pegylated L-asparaginase; TBI, total body irradiation; UKALL, United Kingdom acute lymphoblastic leukemia trial.

*Table note: Each row represents one individual patient unless otherwise specified. Multiple rows with the same Study ID indicate different patients from the same publication. The “Total eligible cases in study” column refers to the number of eligible patients contributed by that study. For Peng et al., individual baseline characteristics were not available; therefore, the row is presented as aggregate cohort-level data.

Patients were heavily pretreated prior to daratumumab. Among evaluable cases, patients had received a median of 1.5 prior systemic therapy lines (range, 1–4), including standard multi-agent ALL regimens, nelarabine-based therapies, venetoclax-containing combinations, targeted agents, and salvage chemotherapy. Several patients had undergone prior allo-HSCT; notably, one patient relapsed after two allogeneic transplants before receiving daratumumab,^23^ and another relapsed with CD38-positive disease six months after a second transplant and subsequently died.^14^

Indications for daratumumab were refractory disease in 8 patients (8/34, 23.5%), overt relapse in 14 (41.2%), and MRD positivity in 12 (35.3%). CD38 expression was assessed in a subset of patients, with nearly all evaluable cases demonstrating CD38-positive blasts, supporting the rationale for daratumumab therapy. In one report, persistent high CD38 expression was documented at MRD-positive relapse, suggesting CD38 stability as a therapeutic target.^7^ For details refer to Table 1.

###  Daratumumab dosing schedule and route of administration

Among 21 patients with documented dosing, intravenous daratumumab at 16 mg/kg was used in 20 patients (95.2%), while one patient (4.8%) received 1,800 mg subcutaneously.^22^ Dosing schedules were frequently extrapolated from myeloma protocols,and in at least one case, combination therapy was discontinued early because of toxicity, with continuation as daratumumab monotherapy.^25^

The number of doses administered was reported in 20 of 33 evaluable cases (60.6%). Patients received a median of 8 doses (IQR, 3–14; range, 2–21), indicating substantial variability in treatment duration.

Protocl data was available for 29 patients. Of these, 23 (79.2%) received daratumumab in combination with other anticancer agents, while 6 (20.8%) received daratumumab monotherapy. Venetoclax-containing regimens were used in 16 patients, including the DV-CAG regimen (daratumumab, venetoclax, cytarabine, aclarubicin, and G-CSF) described by Peng et al.^21^ as a transplant-bridging strategy. Nelarabine-based combinations were used in four patients (16.7%). Other regimens included dara-bortezomib-dexamethasone as a bridge to to haploidentical transplantation^5^ and prolonged daratumumab maintenance after MRD eradication with subsequent donor lymphocyte infusion.^20^ This heterogeneity reflects the absence of a standardized daratumumab-based protocol in R/R T-ALL/T-LBL. For details refer to Table 2.

**Table 2.** Daratumumab treatment characteristics and combination regimens.

No.	Study ID	Patient ID within study	Daratumumab route and dose	Number of daratumumab doses	Combination therapy with daratumumab	Nelarabine included	Venetoclax included	Details of anticancer agents given with daratumumab
1	Prejzner, 2023^22^	Patient 1	IV, 16 mg/kg	3	Yes	Yes	Yes	Nelarabine; venetoclax; bortezomib
2	Prejzner, 2023^22^	Patient 2	SC, 1,800 mg	3	Yes	NR	Yes	Bortezomib; venetoclax; PEG-asparaginase
3	Vakrmanová, 2022^25^	Patient 1	IV, 16 mg/kg	9	Yes	NR	NR	NR
4	Vakrmanová, 2022^25^	Patient 2	IV, 16 mg/kg	21	Yes	NR	NR	NR
5	Vakrmanová, 2022^25^	Patient 3	IV, 16 mg/kg	9	Yes	NR	NR	NR
6	Vakrmanová, 2022^25^	Patient 4	IV, 16 mg/kg	3	No	NR	NR	NR
7	Molle, 2022^19^	Patient 1	IV, 16 mg/kg	13	Yes	Yes	NR	Nelarabine; pegylated asparaginase; dexamethasone
8	Molle, 2022^19^	Patient 2	IV, 16 mg/kg	17	Yes	Yes	NR	Nelarabine; dexamethasone
9	Punatar, 2021^23^	Patient 1	NR	21	Yes	NR	NR	Intrathecal methotrexate; 6-mercaptopurine; oral methotrexate
10	Fulcher, 2021^17^	Patient 1	IV, 16 mg/kg	7	No	NR	NR	None reported
11	Mirgh, 2019^18^	Patient 1	IV, 16 mg/kg	14	Yes	NR	NR	High-dose methotrexate as CNS prophylaxis after dose 8 of daratumumab
12	Mirgh, 2019^18^	Patient 2	IV, 16 mg/kg	2	No	NR	NR	NR
13	Begna, 2024^13^	Patient 1	IV, 16 mg/kg	14	Yes	NR	NR	Brentuximab vedotin
14	Ruhayel, 2021^24^	Patient 1	IV, 16 mg/kg	2	No	NR	NR	NR
15	Bonda, 2018^14^	Patient 1	IV, 16 mg/kg	12	Yes	NR	NR	Intrathecal methotrexate; 6-mercaptopurine; oral methotrexate
16	Ofran, 2020^20^	Patient 1	IV, 16 mg/kg	8	No	NR	NR	NR
17	Ofran, 2020^20^	Patient 2	IV, 16 mg/kg	4	Yes	NR	NR	Donor lymphocyte infusion
18	Castellanos, 2024^15^	Patient 1	NR	NR	Yes	Yes	NR	Nelarabine; radiotherapy
19	Maraglino, 2025^5^	Patient 1	IV, 16 mg/kg	3	Yes	NR	NR	Bortezomib; dexamethasone
20	Kaya, 2025^26^	Patient 1	IV, 16 mg/kg	2	Yes	NR	Yes	Venetoclax; azacitidine; dexamethasone
21	Feng, 2025^7^	Patient 1	NR	NR	Yes	NR	Yes	Venetoclax; azacitidine; homoharringtonine; cytarabine; aclarubicin
22	Dai, 2024^16^	Patient 9	IV, 16 mg/kg	NR	Yes	NR	Yes	DAC plus venetoclax
23	Dai, 2024^16^	Patient 10	IV, 16 mg/kg	NR	Yes	NR	NR	Hyper-CVAD-B plus PEG-asparaginase
24	Peng, 2025^21^	Aggregate cohort	IV, 16 mg/kg	2–4	Yes	NR	Yes	Venetoclax; cytarabine; aclarubicin; G-CSF (DV-CAG regimen)

**Abbreviations:** mg/kg, milligram per kilogram; SC, subcutaneous; mg, milligram; PEG-asparaginase, pegylated asparaginase; CNS, central nervous system; G-CSF, granulocyte colony-stimulating factor; DV-CAG, daratumumab, venetoclax, cytarabine, aclarubicin, and G-CSF; DLI, donor lymphocyte infusion; DAC, decitabine or decitabine-like; VEN, venetoclax; hyper-CVAD-B+PEG-Asp, hyperfractionated cyclophosphamide, vincristine, doxorubicin, and dexamethasone (part A) plus methotrexate and cytarabine (part B) plus PEG-asparaginase; NA, not available.

### Efficacy, survival, and transplantation outcomes

Efficacy outcomes were available for all 34 patients. Complete remission (CR) was achieved in 30/34 patients (88.2%), partial remission (PR) in 2/34 (5.9%), and no response (NR) in 2/34 (5.9%). The Peng et al. cohort contributed 11 CRs.^21^ Notably, Fulcher et al. reported that daratumumab was initially ineffective in the setting of high leukemia burden but subsequently achieved eradication of low-volume residual disease after tumor debulking with a nelarabine-containing salvage regimen.^17^

Among the 30 patients achieving CR, MRD status was reported for 29 patients. Of these, 24/29 (82.8%) achieved MRD negativity and 5/29 (17.2%) remained MRD positive. In the Peng et al. cohort, 9/11 patients (81.8%) achieved MRD negativity, while 2/11 (18.2%) remained MRD positive after daratumumab-based therapy.^21^

The median PFS was 10 months (range, 0.4–45), and the median OS was 10 months (range, 1.5–45). In Peng et al., median OS was not reached after a median follow-up of 15.4 months (range, 1.5–22.4).^21^ These survival estimates should be interpreted cautiously, as many OS and PFS values were derived from last reported follow-up rather than confirmed progression or death events. This likely explains the similarity between OS and PFS, since both endpoints were often represented by the same follow-up duration when separate progression or death dates were unavailable.

In an exploratory treatment-strategy analysis, 6 patients received daratumumab monotherapy and 23 received daratumumab in combination with other anticancer agents. Among patients treated with monotherapy, 1/6 had NR and 1/6 achieved PR; similarly, among those receiving combination therapy, 1/23 had NR and 1/23 achieved PR. No formal statistical comparison was performed because of small sample size, regimen heterogeneity, selection bias, and confounding by indication.

Overall, 18/34 patients (52.9%) proceeded to allogeneic HSCT after daratumumab, including 8/11 patients from the Peng et al. cohort,^21^ while 16/34 (47.1%) did not. Daratumumab was frequently used as a bridge to transplant, including in patients with refractory disease or early post-transplant relapse. Complex transplant histories were observed, including two patients who had undergone two prior allo-HSCTs before daratumumab therapy.^14,23^ Among transplanted patients, 13/18 (72.2%) were alive at last follow-up and 5/18 (27.8%) had died. The median post-HSCT survival/follow-up was 8.6 months (IQR, 4.25–14.5; range, 1–42), with a mean of 11.4 months. Among patients who did not proceed to HSCT, 11/16 (68.8%) were alive and 5/16 (31.2%) had died at last follow-up.

Among the 10 reported deaths in the overall cohort, causes included disease progression or relapse in 7/10 (70%), septic shock in 1/10 (10%), GVHD in 1/10 (10%), and ARDS in 1/10 (10%). These transplant-related findings suggest that daratumumab may facilitate bridging to HSCT in selected patients; however, they should not be interpreted as a direct comparison between post-daratumumab HSCT and non-HSCT strategies, as transplant receipt was likely influenced by response, clinical fitness, donor availability, disease control, and incomplete patient-level reporting. Detailed outcomes are provided in Tables 3 and 4.

**Table 3.** Response, survival, transplantation, and safety outcomes after daratumumab-based therapy.

No.	Study ID	Patient ID within study	Response	MRD status after daratumumab	OS/PFS after daratumumab start, months	Proceeded to allo-HSCT after daratumumab	Survival after allo-HSCT, months	Outcome after HSCT	Cause of death	Infusion reaction	Cytopenia	Infection
1	Prejzner, 2023^22^	Patient 1	CR	Positive	11	Yes	10	Alive	NR	NR	Yes	NR
2	Prejzner, 2023^22^	Patient 2	CR	Positive	NR	Yes	10	Alive	NR	NR	Yes	NR
3	Vakrmanová, 2022^25^	Patient 1	CR	Negative	11	Yes	7	Died	Disease relapse	NR	NR	NR
4	Vakrmanová, 2022^25^	Patient 2	CR	Negative	NR	Yes	5	Died	Disease relapse	NR	NR	NR
5	Vakrmanová, 2022^25^	Patient 3	CR	Negative	NR	Yes	15	Alive	NR	NR	NR	NR
6	Vakrmanová, 2022^25^	Patient 4	NR	Positive	1.7	No	NR	NR	Disease progression	NR	NR	NR
7	Molle, 2022^19^	Patient 1	CR	NR	45	Yes	42	Alive	NR	NR	Yes	NR
8	Molle, 2022^19^	Patient 2	CR	Negative	11.5	No	NR	NR	Disease progression	NR	Yes	Yes
9	Punatar, 2021^23^	Patient 1	CR	Negative	42	No	NR	NR	NR	Yes, grade 3	NR	Yes
10	Fulcher, 2021^17^	Patient 1	CR	Negative	5.2	Yes	4	Alive	NR	NR	NR	NR
11	Mirgh, 2019^18^	Patient 1	CR	Negative	NR	Yes	1	Died	Septic shock	NR	NR	NR
12	Mirgh, 2019^18^	Patient 2	CR	Negative	10.7	No	NR	NR	NR	NR	NR	NR
13	Peng, 2025^21^	Aggregate cohort	CR, 11/11	Negative, 9/11	Median OS not reached; median follow-up 15.4 months, range 1.5–22.4	Yes, 8/11	NR	NR	NR	Yes, 7 patients, grade 1–2	Yes, 7 patients, grade 3–4	NR
14	Peng, 2025^21^	Patient 1	CR	Negative	22	Yes	20	Alive	NR	NR	Yes	Yes
15	Peng, 2025^21^	Patient 2	CR	Positive	16.5	Yes	3.5	Died	Disease relapse	NR	NR	NR
16	Peng, 2025^21^	Patient 3	CR	Negative	19	Yes	18	Alive	NR	NR	NR	Yes
17	Peng, 2025^21^	Patient 4	CR	Negative	15.5	Yes	12.5	Alive	NR	NR	NR	NR
18	Peng, 2025^21^	Patient 5	CR	Negative	15	Yes	13	Alive	NR	NR	NR	NR
19	Peng, 2025^21^	Patient 6	CR	Negative	7	Yes	5.5	Died	Graft-versus-host disease	NR	NR	NR
20	Peng, 2025^21^	Patient 7	CR	Negative	10	Yes	1	Alive	NR	Yes, grade 1	Yes	Yes
21	Peng, 2025^21^	Patient 8	CR	Negative	4	Yes	1.5	Alive	NR	NR	Yes	Yes
22	Peng, 2025^21^	Patient 9	CR	Negative	4	No	NR	NR	NR	Yes, grade 1	Yes	NR
23	Begna, 2024^13^	Patient 1	CR	Negative	8	No	NR	NR	NR	NR	NR	NR
24	Ruhayel, 2021^24^	Patient 1	PR	NR	1.4	No	NR	NR	Disease progression	NR	NR	NR
25	Bonda, 2018^14^	Patient 1	CR	Negative	5	No	NR	NR	NR	NR	NR	NR
26	Ofran, 2020^20^	Patient 1	CR	Negative	10	No	NR	NR	NR	NR	NR	NR
27	Ofran, 2020^20^	Patient 2	CR	Negative	10.5	No	NR	NR	NR	NR	NR	NR
28	Castellanos, 2024^15^	Patient 1	PR	NR	1.5	No	NR	NR	Disease progression	NR	NR	NR
29	Maraglino, 2025^5^	Patient 1	CR	Negative	31	Yes	29	Alive	NR	NR	NR	NR
30	Kaya, 2025^26^	Patient 1	CR	NR	NR	No	NR	NR	ARDS	NR	NR	NR
31	Feng, 2025^7^	Patient 1	CR	Negative	10	Yes	7.2	Alive	NR	NR	NR	NR
32	Dai, 2024^16^	Patient 9	NR	Positive	NR	No	NR	NR	NR	NR	NR	NR
33	Dai, 2024^16^	Patient 10	CR	Negative	NR	No	NR	NR	NR	NR	NR	NR

**Abbreviations:** CR, complete response; PR, partial response; NR, no response/not reported; MRD, minimal residual disease; OS/PFS, overall survival/progression-free survival; Dara, daratumumab; HSCT, hematopoietic stem cell transplantation; ARDS, acute respiratory distress syndrome; NA, not available.

*Table note: Each row represents one individual patient unless otherwise specified. Multiple rows with the same Study ID indicate different patients from the same publication. For Peng et al., both aggregated cohort-level data and available individual patient-level outcomes are shown because some outcomes were reported in aggregate and others were extractable at the patient level.

**Table 4.** Summary of efficacy, survival, and transplantation outcomes following daratumumab-based therapy (N = 34).

Outcome category	Result
**Patients evaluated for efficacy**	34
**Best overall response**	
Complete remission (CR)	30/34 (88.2%)
Partial remission (PR)	2/34 (5.9%)
No response (NR)	2/34 (5.9%)
**Complete responders with MRD assessment**	29
MRD negative	24/29 (82.8%)
MRD positive	5/29 (17.2%)
**Median PFS**	10 months (range, 0.4–45)
**Median OS**	10 months (range, 1.5–45)
**Patients proceeding to allo-HSCT**	18/34 (52.9%)
**Patients not proceeding to allo-HSCT**	16/34 (47.1%)
**Post-HSCT outcomes**	
Alive at last follow-up	13/18 (72.2%)
Deceased	5/18 (27.8%)
**Median post-HSCT survival/follow-up**	8.6 months (range, 1–42)

**Abbreviations:** OS, overall survival; PFS, progression-free survival; MRD, measurable residual disease; HSCT, hematopoietic stem cell transplantation.

### Safety

Because most patients received daratumumab in combination with multi-agent chemotherapy, attribution of cytopenia and infectious complications specifically to daratumumab was challenging. Several reports suggested that adverse events were more likely to be related to concomitant regimens or cumulative toxicity from prior therapies. In contrast, infusion-related reactions were clearly attributable to daratumumab, occurring in 10 patients, the majority of which were grade 1–2 (9/10, 90%). A grade 3 infusion reaction was reported in one patient,^23^ leading to treatment discontinuation in that case, and no grade 4 reactions were observed. For details refer to Table 3

## Discussion

This systematic review synthesizes the currently available evidence on daratumumab-based therapy in R/R T-ALL/T-LBL. Across 16 publications and 34 patients, we observed a high overall response rate, with 88.2% achieving complete remission (CR), substantial clearance of measurable residual disease (MRD), meaningful post-transplant survival in a heavily pretreated population, and a safety profile dominated by low grade infusion reactions. These findings support CD38 targeting as a rational and clinically active strategy in R/R T ALL/T LBL, a setting in which outcomes with conventional salvage therapy remain dismal.

Adult patients with R/R T-ALL/T-LBL experience very poor outcomes, with historical complete remission (CR) rates to first salvage of approximately 40–46% and median overall survival of 6–9 months, even with intensive chemotherapy and allogeneic transplantation.^27–29^ In the phase 2 Delphinus study evaluating daratumumab as part of combination therapy in pediatric and young adult patients with R/R T-ALL/T-LBL (pediatric T-ALL = 24; young adult T-ALL = 5; pediatric or young adult T-LBL = 10), the best overall response rate was 74%, with a CR rate of 49%.^3^

In this context, the 88.2% CR rate and 5.9% partial remission rate observed in our pooled cohort compare favorably with established benchmarks, particularly given the heavy pretreatment history of most patients and the high proportion with prior allogeneic transplantation. Although these data derive from uncontrolled reports on a highly selected patient population, the magnitude of response suggests that daratumumab-based regimens can induce deep remissions in a population otherwise characterized by chemotherapy resistance.

The biological rationale for CD38 targeting T-ALL/T-LBL is strong. Leukemic blasts express high and relatively homogeneous levels of CD38, and expression persists following multi-agent chemotherapy.^8^ CD38 expression on normal hematopoietic cells is comparatively low, creating a therapeutic window for antibody-based strategies. Daratumumab, a human IgG1κ monoclonal antibody, induces tumor cell death through complement-dependent cytotoxicity, antibody-dependent cellular cytotoxicity, antibody-dependent cellular phagocytosis, and direct apoptotic signaling.^30^ Preclinical models have demonstrated potent daratumumab activity against CD38-positive T-ALL/T-LBL samples in vitro and in xenograft systems.

MRD status is now recognized as one of the most powerful prognostic factors in both adult and pediatric acute lymphoblastic leukemia, with MRD negativity consistently associated with lower relapse risk and improved long-term survival.^31^ In T-ALL specifically, MRD positivity at key treatment landmarks and at the time of transplantation correlates with substantially higher relapse rates and inferior event-free survival.^32^ In the Delphinus trial, 12 of the 29 patients with T-cell acute lymphoblastic leukemia—representing 41% of the total population and 50% of responding patients—achieved MRD-negative status.^3^

Against this background, the finding that 82.8% of evaluable patients in our cohort achieved MRD negativity following daratumumab-based therapy suggests that CD38 targeting may be particularly effective as a debulking or MRD-eradicating strategy before allogeneic transplantation. This aligns with broader transplant literature showing superior outcomes in MRD-negative compared with MRD-positive patients undergoing allogeneic transplantation for acute lymphoblastic leukemia.^33^ Although our review cannot definitively link MRD conversion to survival because of heterogeneous reporting, the observed pattern of frequent MRD negativity, successful transition to transplantation, and favorable post-transplant status in several patients supports the potential role of daratumumab as a bridge to transplantation in appropriately selected cases.

Most patients in our review received daratumumab alongside other cytotoxic or targeted agents, reflecting contemporary practice in which single-agent antibody therapy is rarely used in R/R T-ALL/LBL. Venetoclax-based combinations were particularly frequent, including multi-agent regimens such as DV-CAG (daratumumab, venetoclax, cytarabine, aclarubicin, and G-CSF). This observation aligns with emerging evidence showing meaningful activity of venetoclax—especially when combined with azacitidine, cladribine-based regimens, or CHG-like backbones in R/R T-ALL/LBL.^34^

In the Delphinus trial, daratumumab was administered in combination with multiple chemotherapy agents, reflecting an intensive multi-agent strategy. The phase 2 DELPHINUS study evaluated daratumumab plus vincristine/prednisone/pegylated-asparaginase/doxorubicin reinduction in childhood and young adult T-cell ALL/LL in first relapse.^3^ This protocol illustrates the breadth of cytotoxic and antimetabolite partners used alongside daratumumab. Additional retrospective evidence also supports the feasibility of daratumumab in combination with chemotherapy.^35^ Interestingly, several reports documented complete remissions with daratumumab monotherapy in patients who were otherwise refractory to prior chemotherapy.^17,18,20^ These cases suggest that daratumumab may exert direct anti-leukemic activity independent of combination partners.

Given the multifactorial nature of these regimens, the relative contribution of daratumumab versus the chemotherapy backbone cannot be fully delineated. Nevertheless, the consistently high complete remission and MRD-negative rates across diverse daratumumab-containing combinations—together with preclinical evidence of CD38-directed cytotoxicity—support the hypothesis that daratumumab functions as an active therapeutic component rather than a passive adjunct. Prospective studies will be required to determine whether daratumumab provides additive or synergistic benefit beyond optimized venetoclax- or nelarabine-based regimens.

Despite the heavily pretreated nature of the cohort, the median PFS and OS were both 10 months, with wide ranges, suggesting that some patients experienced clinically meaningful disease control after daratumumab-based therapy. These findings compare favorably with historical outcomes in adult R/R T-ALL/T-LBL, where median overall survival after first salvage is approximately 6 months and long-term survival remains uncommon. However, these survival estimates should be interpreted cautiously because many values were derived from last reported follow-up rather than mature time-to-event data. In the DELPHINUS trial, median event-free survival was 8.9 months for childhood T-ALL, 10.3 months for young adult T-ALL, 9.5 months for the pooled population, and 2.9 months for T-LBL. By contrast, in a small retrospective study of 20 patients with R/R ALL, including 10 with R/R T-ALL, treated with daratumumab-based therapy, the median overall survival was only 4 weeks and the complete remission rate was 20%, underscoring the aggressive biology and heterogeneity of outcomes in this setting.^35^

The safety profile observed in this review is broadly consistent with the established experience of daratumumab in multiple myeloma and other CD38-expressing hematologic malignancies. No new safety signals were identified. This pattern mirrors prior observations in myeloma, where most infusion reactions occur during the first infusion and are generally manageable with premedication and supportive care.^36^

### Strengths

This review has several strengths. To our knowledge, it represents one of the most comprehensive syntheses focused specifically on daratumumab-based regimens in R/R T-ALL and T-LBL, extending earlier reviews that included mixed T-lineage and non–T-lineage acute leukemias or pre-2023 data. We systematically characterized response rates, MRD outcomes, transplant utilization, post-HSCT outcomes, and toxicity, providing a multidimensional overview of the current clinical role of daratumumab in this setting. In addition, the review captures a broad spectrum of real-world practice across multiple regions and time periods, reflecting the diversity of salvage strategies used in routine care and early-phase clinical research.

### Future directions

Our findings support further exploration of rational daratumumab-based combinations, particularly with venetoclax, nelarabine, and other agents with established activity in T-ALL/T-LBL, to enhance response depth and durability while minimizing overlapping toxicity. Prospective multicenter trials are needed to validate these observations, define optimal dosing and scheduling, and clarify the role of daratumumab relative to other emerging immunotherapeutic approaches. Notably, prospective evaluation in the MRD-positive setting is already underway. The T-ALL-2024 trial (NCT06570915) is a prospective, single-arm, multicenter, open-label phase II study evaluating the efficacy and safety of daratumumab in adult patients with T-ALL who remain measurable residual disease-positive after standard chemotherapy. By specifically targeting patients with flow cytometry-detectable MRD, this trial may help determine whether daratumumab is best positioned as an MRD-directed strategy rather than solely as salvage therapy for overt relapsed or refractory disease. Future studies incorporating standardized MRD assessment, uniform response criteria, and predefined transplant strategies will be essential to determine whether daratumumab improves long-term, transplant-adjusted outcomes.

### Limitations

Important limitations should temper the interpretation of these findings. All included reports were observational and consisted predominantly of single-patient case reports or very small case series, making the analysis highly vulnerable to selection and publication bias. In particular, the high rates of complete remission and MRD negativity should not be interpreted as definitive estimates of treatment efficacy, because the denominator reflects published cases rather than all patients treated with daratumumab in clinical practice. Favorable or unusual responses are more likely to be reported, whereas non-response, early progression, or treatment-related complications may be underrepresented. The small sample size, heterogeneous treatment regimens, variable response assessment methods, inconsistent survival reporting, and limited follow-up further preclude robust subgroup analyses or pooled survival estimates. Therefore, these findings should be viewed as hypothesis-generating and require validation in prospective, systematically enrolled cohorts.

## Conclusion

Daratumumab-based regimens may induce deep remissions and MRD negativity in selected patients with CD38-positive relapsed or refractory T-ALL/T-LBL and may facilitate bridging to allogeneic HSCT in some cases. However, these findings should be interpreted cautiously given the small sample size, predominance of case reports, and risk of publication bias. Prospective studies are needed to validate the efficacy, safety, and optimal positioning of daratumumab within salvage treatment algorithms.

## Supplemental material


Supplemental Material - Targeting CD38 in relapsed or refractory T-cell acute lymphoblastic leukemia/lymphoma: A systematic review of daratumumab-based therapy
Supplemental Material for Targeting CD38 in relapsed or refractory T-cell acute lymphoblastic leukemia/lymphoma: A systematic review of daratumumab-based therapy by Abdulrahman F. Al-Mashdali, Mujahid O. Abdelraof, Monia Ibrahim, Mohammed Abdulgayoom, Shehab F. Mohamed and Honar Cherif in Therapeutic Advances in Hematology.

Supplemental Material - Targeting CD38 in relapsed or refractory T-cell acute lymphoblastic leukemia/lymphoma: A systematic review of daratumumab-based therapy
Supplemental Material for Targeting CD38 in relapsed or refractory T-cell acute lymphoblastic leukemia/lymphoma: A systematic review of daratumumab-based therapy by Abdulrahman F. Al-Mashdali, Mujahid O. Abdelraof, Monia Ibrahim, Mohammed Abdulgayoom, Shehab F. Mohamed and Honar Cherif in Therapeutic Advances in Hematology.

Supplemental Material - Targeting CD38 in relapsed or refractory T-cell acute lymphoblastic leukemia/lymphoma: A systematic review of daratumumab-based therapy
Supplemental Material for Targeting CD38 in relapsed or refractory T-cell acute lymphoblastic leukemia/lymphoma: A systematic review of daratumumab-based therapy by Abdulrahman F. Al-Mashdali, Mujahid O. Abdelraof, Monia Ibrahim, Mohammed Abdulgayoom, Shehab F. Mohamed and Honar Cherif in Therapeutic Advances in Hematology.

## Data Availability

All data supporting the findings of this study are included within the article and its supplementary materials. Additional information is available from the corresponding author upon reasonable request.*
