# Supplementary material for: Targeting CD38 in relapsed /refractory T-cell acute lymphoblastic leukemia/lymphoma: A systematic review of daratumumab-based therapy
Source: Ther Adv Hematol. 2026 Aug 25;17:20406207261484097. doi: 10.1177/20406207261484097 (PMC13519216; doi:10.1177/20406207261484097)
Supplement: Supplementary file 1 [file sj-pdf-1-tah-10.1177_20406207261484097.pdf]

Supplementary Methods: A Priori Protocol

Protocol Development and Reporting Standards

An a priori protocol was developed prior to study initiation to define the research question, eligibility criteria, outcomes of interest, and analytical approach. Although the protocol was not prospectively registered in PROSPERO, all methodological steps were predefined and adhered to throughout the review process. The study was conducted and reported in accordance with the **Preferred Reporting Items for Systematic Reviews and Meta-Analyses (PRISMA 2020)** guidelines.

Research Question and Framework

This systematic review aimed to evaluate the **efficacy and safety of daratumumab-based therapy in patients with relapsed or refractory (R/R) T-cell acute lymphoblastic leukemia/lymphoma (T-ALL/T-LBL)**.

The review was structured according to a modified **PICOS framework**:

- **Population:** Patients with R/R T-ALL or T-LBL (all age groups)
- **Intervention:** Daratumumab administered as monotherapy or in combination with other agents
- **Comparator:** Not required
- **Outcomes:** Clinical response, MRD status, survival outcomes (PFS, OS), transplantation outcomes, and safety
- **Study design:** Observational studies, case series, and case reports

Eligibility Criteria

Inclusion Criteria

Studies were eligible if they:

1. Included patients diagnosed with **R/R T-ALL or T-LBL**
2. Reported treatment with **daratumumab-based regimens**
3. Provided **extractable clinical data at the individual or cohort level**
4. Reported at least one outcome of interest (efficacy, survival, or safety)
5. Were published in **English**

Exclusion Criteria

Studies were excluded if they:

- Focused on **newly diagnosed disease only**
- Included **non–T-cell malignancies without extractable subgroup data**
- Were **reviews, editorials, letters without data, laboratory-only, or animal studies**
- Lacked sufficient clinical detail for data extraction

Conference abstracts were included when sufficient data were available.

Information Sources and Search Strategy

A systematic literature search was conducted in PubMed/MEDLINE, Scopus, and Web of Science from database inception through December 1, 2025. The search combined controlled vocabulary terms and free-text keywords related to T-cell acute lymphoblastic leukemia/lymphoma, including "T-ALL" and "T-LBL," and CD38-directed therapy, including "daratumumab" and "CD38."
In addition, relevant conference proceedings from ASH, EHA, and ASCO were manually searched using combinations of the same keywords to identify eligible abstracts not captured in database searches. Reference lists of included studies and relevant review articles were also screened to identify additional reports.

Study Selection Process

All records were imported into a reference management system and duplicates were removed.

- **Step 1:** Two reviewers independently screened titles and abstracts
- **Step 2:** Full-text assessment of potentially eligible studies
- **Step 3:** Discrepancies were resolved by consensus or third-reviewer adjudication

A PRISMA flow diagram summarizes the selection process.

Data Extraction

Data were extracted independently by two reviewers using a **standardized electronic data collection form**.

The following variables were collected:

- Study characteristics (year, country, design)
- Patient demographics and disease features
- Prior therapies and transplant history

- Daratumumab regimen (dose, route, combinations)
- CD38 expression
- **Efficacy outcomes:** CR, PR, NR, MRD status
- **Survival outcomes:** PFS, OS
- **Transplant outcomes:** bridging to allo-HSCT and post-transplant survival
- **Safety outcomes:** infusion reactions, cytopenias, infections

Discrepancies in extraction were resolved by consensus.

Outcome Definitions

To ensure consistency across heterogeneous reports, the following operational definitions were applied:

- **Complete remission (CR), partial remission (PR), and no response (NR):** Defined according to the criteria reported in individual studies
- **MRD negativity:** Defined based on study-specific thresholds
- **Progression-Free Survival (PFS):**
  Time from initiation of daratumumab to **documented disease progression, relapse, death, or last follow-up**
- **Overall Survival (OS):**
  Time from initiation of daratumumab to **death from any cause or last follow-up**

For individual case reports:

- When progression timing was not explicitly reported, **death was considered the PFS event**
- Patients with **primary refractory disease** were classified as having progressive disease, with PFS measured to the time of progression or death rather than assigned a value of zero

Quality Assessment

Risk of bias was assessed according to study design:

- **Case reports and case series:** Murad et al. tool (selection, ascertainment, causality, reporting)
- **Observational cohort studies:** Newcastle-Ottawa Scale (NOS)

Quality assessment was performed independently by two reviewers.

Data Synthesis and Statistical Analysis

Given the anticipated heterogeneity and predominance of small observational studies:

- A **quantitative meta-analysis was not performed**
- Data were synthesized using a **descriptive and narrative approach**

Analytical methods included:

- Continuous variables: reported as **median (range or IQR)**
- Categorical variables: reported as **frequency and percentage**
- Response rates calculated across the pooled population
- Subgroup summaries performed where feasible

Survival outcomes (PFS and OS) were summarized descriptively due to:

- Variable reporting formats
- Small sample size
- Lack of uniform censoring information

All analyses were conducted using standard spreadsheet software.

Ethics and Data Availability

This study utilized **published, de-identified data only**; therefore, ethical approval was not required. Extracted datasets and supplementary materials are available upon reasonable request.
