# Supplementary material for: Targeting CD38 in relapsed /refractory T-cell acute lymphoblastic leukemia/lymphoma: A systematic review of daratumumab-based therapy
Source: Ther Adv Hematol. 2026 Aug 25;17:20406207261484097. doi: 10.1177/20406207261484097 (PMC13519216; doi:10.1177/20406207261484097)
Supplement: Supplementary file 2 [file sj-pdf-2-tah-10.1177_20406207261484097.pdf]

Supplementary Table 1. NOS for the risk of bias and quality assessment of Cohort studies

Author	Year	Selection				Comparability	Outcome			Total score
		Representativeness of the exposed cohort	Selection of the non-exposed cohort	Ascertainment of exposure	Demonstration that outcome of interest was not present at start	Comparability of cohorts on the basis of design or analysis	Assessment of outcome	Was follow-up long enough for outcomes to occur?	Adequacy of follow-up of cohorts	
Dai et al.	2024	⋆	None	⋆⋆	⋆	None	⋆	⋆	⋆	7

NOS, Newcastle-Ottawa scale.

Supplementary Table 2. Murad et al. methodological quality tool for case reports and case series

**Study**	**Year of publication**	**Type of publication**	**Does the patient(s) represent(s) the whole experience of the investigator (center) or is the selection method unclear to the extent that other patients with similar presentation may not have been reported?**	**Was the exposure adequately ascertained?**	**Was the outcome adequately ascertained?**	**Was follow-up long enough for outcomes to occur?**	**Is the case(s) described with sufficient details to allow other investigators to replicate the research or to allow practitioners make inferences related to their own practice?**	**Overall judgment**
Feng	2025	Case report	N	Y	Y	Y	N	Acceptable methodological quality
kaya	2025	Case report	N	Y	Y	?	Y	Acceptable methodological quality
Maraglino	2025	Case report	N	Y	Y	Y	Y	Acceptable methodological quality
Castellanos	2024	Case report	N	Y	Y	Y	Y	Acceptable methodological quality
Ofran	2020	Case series	N	Y	Y	Y	Y	Acceptable methodological quality
Bonda	2018	Case report	N	Y	Y	Y	Y	Acceptable methodological quality
Ruhaye	2021	Case report	N	Y	N	Y	Y	Acceptable methodological

								quality
Begna	2024	Case report	N	Y	Y	Y	Y	Acceptable methodological quality
Peng	2025	Case series	N	Y	Y	Y	Y	Acceptable methodological quality
Mirgh	2019	Case series	N	Y	Y	Y	Y	Acceptable methodological quality
Fulcher	2021	Case report	N	Y	Y	Y	Y	Acceptable methodological quality
Punatar	2021	Case report	N	Y	Y	Y	Y	Acceptable methodological quality
Molle	2022	Case series	N	Y	Y	Y	Y	Acceptable methodological quality
Vakrmanov á	2022	Case series	N	Y	Y	Y	Y	Acceptable methodological quality
Prejzner	2023	Case series	N	Y	Y	Y	Y	Acceptable methodological quality
