# Supplementary material for: Targeting CD38 in relapsed /refractory T-cell acute lymphoblastic leukemia/lymphoma: A systematic review of daratumumab-based therapy
Source: Ther Adv Hematol. 2026 Aug 25;17:20406207261484097. doi: 10.1177/20406207261484097 (PMC13519216; doi:10.1177/20406207261484097)
Supplement: Supplementary file 3 [file sj-pdf-3-tah-10.1177_20406207261484097.pdf]

Database	Search strategy
**PubMed/MEDLINE**	(("daratumumab"[Title/Abstract] OR "anti-CD38"[Title/Abstract] OR "CD38"[Title/Abstract]) AND ("T-cell acute lymphoblastic leukemia"[Title/Abstract] OR "T cell acute lymphoblastic leukemia"[Title/Abstract] OR "T-ALL"[Title/Abstract] OR "T-cell acute lymphoblastic lymphoma"[Title/Abstract] OR "T cell acute lymphoblastic lymphoma"[Title/Abstract] OR "T-lymphoblastic lymphoma"[Title/Abstract] OR "T-LBL"[Title/Abstract] OR "lymphoblastic leukemia"[Title/Abstract] OR "lymphoblastic lymphoma"[Title/Abstract]))
**Scopus**	TITLE-ABS-KEY (daratumumab OR "anti-CD38" OR CD38) AND TITLE-ABS-KEY ("T-cell acute lymphoblastic leukemia" OR "T cell acute lymphoblastic leukemia" OR "T-ALL" OR "T-cell acute lymphoblastic lymphoma" OR "T cell acute lymphoblastic lymphoma" OR "T-lymphoblastic lymphoma" OR "T-LBL" OR "lymphoblastic leukemia" OR "lymphoblastic lymphoma")
**Web of Science**	TS=(daratumumab OR "anti-CD38" OR CD38) AND TS=("T-cell acute lymphoblastic leukemia" OR "T cell acute lymphoblastic leukemia" OR "T-ALL" OR "T-cell acute lymphoblastic lymphoma" OR "T cell acute lymphoblastic lymphoma" OR "T-lymphoblastic lymphoma" OR "T-LBL" OR "lymphoblastic leukemia" OR "lymphoblastic lymphoma")
